# Guano morphology has the potential to inform conservation strategies in British bats

**DOI:** 10.1371/journal.pone.0230865

**Published:** 2020-04-09

**Authors:** Roselyn L. Ware, Benjamin Garrod, Hannah Macdonald, Robin G. Allaby

**Affiliations:** School of Life Sciences, University of Warwick, Coventry, England; Institute of Geographic Sciences and Natural Resources Research Chinese Academy of Sciences, CHINA

## Abstract

Bats are primary consumers of nocturnal insects, disperse nutrients across landscapes, and are excellent bioindicators of an ecosystem’s health, however four of the seventeen Great British species are listed as declining. In this study we aim to investigate the link between bat guano morphology and diet, specifically looking at the ability to predict 1) species, 2) dietary guild, and 3) bat size, using guano morphology alone. Guano from 16 bat species sampled from across Great Britain were analysed to determine various morphological metrics. These data were coupled with diet data obtained by an extensive literature review. It was found that guano morphology overlapped too much to make predictions on the species of bat which deposited the guano, however, in some cases, it could be used to indicate the dietary guild to which the bat belonged. In general, guano morphology seems more correlated to diet than species. This enables the identification of the most important prey taxa within a local environment; a crucial step for informing conservation strategies.

## Introduction

Bats (order Chiroptera) are the second largest group of mammals. Bats are pivotal to supporting global biodiversity; they are the primary consumers of nocturnal insects, disperse nutrients across landscapes, and are excellent bioindicators of an ecosystem’s health [1–3]. However, 25% of bats worldwide are classed as ‘of conservation concern’, with a further 21% classified as ‘near threatened’ [4, 5]. Four of the seventeen Great British species are listed as declining (*Barbastella barbastellus*, *Rhinolophus hipposideros*, *Rhinolophus ferrumequinum*, *and Myotis bechsteinii*), with the status of several others unknown (*Eptesicus serotinus*, *Myotis mysticinus*, *Nyctalus leisleri*, *Nyctaus noctule*, *Pipistrellus nathusii*, *Pipistrellus Pygmaeus*, *and Myotis alcathoe)* [6]. The plethora of threats faced by bats include (but are not limited to): unsympathetic development projects, destruction of tree lines and hedgerows, the drainage of wetlands, infectious diseases, and the impact of pesticides [5, 7]. Additionally, climate change may have a highly detrimental impact on bats, including changes in prey abundances, alterations in the efficacy of echolocation calls, and the consequences of extreme weather events [8, 9]. This is why it is vital to understand their ecological niches and correctly identify species.

Direct observation of predation of insects by bats can be difficult [10]. As a result, analysis of bat diets has relied heavily on microscopic analysis of digested insect fragments found in guano [11]. However, bats thoroughly masticate and digest their prey, often discarding the harder to digest fragments such as the carapace or elytra [12–14]. This increases the likelihood of miss-identification, and over representation of the tougher remains that were not discarded. Identifications made in this manner are rarely more specific than order level [10].

Using bat guano to detect an individual’s diet using either the stable isotope method or the molecular approach has been well established in its accuracy and application [10, 15, 16]. However, it does involve a large budget and in-depth knowledge of the subject. In this study, we wished to investigate whether any potentially useful ecological information, such as diet, could be determined by studying guano morphology alone. The hypothesis being that different dominant prey species may influence the size and shape of the guano produced. To test this hypothesis, we compiled diets from all 17 bat British bat species (*Barbastella barbastellus*, *Eptesicus serotinus*, *Myotis bechsteinii*, *Myotis brandtii*, *Myotis daubentonii*, *Myotis mystacinus*, *Myotis nattereri*, *Nyctalus leisleri*, *Nyctalus noctula*, *Plecotus auritus*, *Plectotus austriacus*, *Pipistrellus nathusii*, *Pipistrellus pipistrellus*, *Pipistrellus pygmaeus*, *Rhinolophus ferrumequinum*, *Rhinolophus hipposideros* and *Myotis alcathoe*) and compared this with a data set of guano morphology. In 1997, Vaughan undertook a review of all of the published diets of the 15 species of bats then known to be present in Great Britain [17]. Since then, two new species of bat have been described, and have been identified to be present in Great Britain: *P*. *pygmaeus* and *M*. *alcathoe*. Furthermore, numerous additional studies have since been published. It was, therefore, deemed valuable to produce an up-to-date synthesis all of the data pertaining to the diets of Great British bats.

In this study we investigate guano morphology data’s ability to identify 1) species, 2) dietary guilds and 3) bat size. Information on species, dietary guild, or size may be used to give ecologists some useful information in the field about bat ecology. Understanding dietary overlaps, and the basis of the diets, will be valuable in helping to direct conservation efforts, and in explaining inter-species interactions [2, 18].

When taking into consideration the density (i.e. where the majority of the samples fall) and the limitations of the study, it may be possible that this data could be used to predict the dietary guild and general size of a bat. This may have applications within outreach or preliminary ecological assessment for field ecologists and be used to potentially identify certain characteristics of the environment, for example, the morphology of a guano sample could be used indicate which invertebrate groups are important to the particular bat in question.

Predicting the dietary guild of a species present in an area is interesting and informative because typically, the most important factors in bat niche separation are considered to be the partitioning of habitat and diet [7, 19]. The ranges of many of the Great British bat species are acutely overlapping [20], suggesting that trophic resource partitioning is important in supporting the species in Great Britain [21, 22]. Prey availability may, in some circumstances, be a more important determiner of species ability to co-inhabit in an area than dietary differences [23]. If a prey taxon is rare, it may become a limiting factor, thus introducing competition between those predating upon it. If a prey taxon is common, competitive exclusion can cause one bat species to be excluded [24].

In general, bats with a higher extinction risk are those with greater dietary specialisation [4, 25]. Where a bat has a narrow dietary range, it is considered more vulnerable to extinction, whereas a bat with a broad dietary breadth is considered to be more robust. Knowledge of the level of dietary specialisation of a species, coupled with intelligence of the conservation status of the prey, is a valuable resource.

## Materials and methods

### Diet

The diet data used in this study was collected from a comprehensive search of the available literature, searching exclusively for bats present in Britain (*B*. *barbastellus*, *E*. *serotinus*, *M*. *bechsteinii*, *M*. *brandtii*, *M*. *daubentonii*, *M*. *mystacinus*, *M*. *nattereri*, *N*. *leisleri*, *N*. *noctula*, *P*. *auritus*, *P*. *austriacus*, *P*. *nathusii*, *P*. *pipistrellus*, *P*. *pygmaeus*, *R*. *ferrumequinum*, *R*. *hipposideros*, and *M*. *alcathoe*). Google Scholar and the Web of Knowledge databases were mined for published bat diets up to January 2016 (Scopus was not used as it does not index many papers older than 1970 [26]). The terms searched for using the Boolean “AND” were: “Diet”, “Food”, and “Prey”, with “Bat”, “Chiroptera”, and the names of each of the species. In some cases, it was appropriate to exclude certain studies. Typically, this was due to the reporting of diets with only presence/absence data, as it was not possible to convert these to numerical data without the introduction of bias. Papers were also excluded if they did not detail diet break down, if they described controlled experiments (i.e. captive fed bats), if stable isotope analysis was used, or they did not use primary data.

In total, we identified 80 published studies, many of which used different methods of determining diet (described in S1 Table), this resulted in a dataset of 212 diets spread across 17 species. For more information regarding the diets, including country of sample collection, references, the study method, and the collated literature data, please see (S2 Table). For the purpose of this study, the data are expressed in number of diets rather than number of studies, as each study may present more than one diet. Each individual reference may present the diets of different species, present the diets of one species measured in different ways, or diets of one species from different sampling locations or seasons. Prey taxa were grouped by order (where possible), class, phylum, or kingdom (where necessary). The mean of all of the diets for each species was calculated. When directly comparing with guano morphology *M*. *alcathoe* was dropped from the dataset as there was no guano samples available with which to compare. Once a count of the taxa identified by each study had been recorded for each study, the results of each publication were collapsed to order level to allow comparison of the studies (S2 Table).

### Guano morphology

Samples were submitted to the University of Warwick as part of the EcoWarwicker Ecological Forensics service, with those collecting the guano responsible for obtaining all necessary permits. All the work described was approved by the University Genetic Modification and Biosafety Committee and the ethical issues were approved by the University Animal Welfare & Ethical Review Body Committee. Anonymised guano samples were obtained with the consent of EcoWarwicker Ecological Forensics. No animals were handled or disturbed in the completion of this project. To ensure that the samples were as representative of each species as possible, samples were selected to cover the whole of Great Britain (as far as the range of the species allowed). Locations are not included here in order to preserve the anonymity of the samples, and no analysis using location information was undertaken.

In addition to Stebbings’ diagnostic characteristics of length (minimum–maximum within a sample), diameter (minimum to maximum) and particle size [27]; we measured colour, and presence/absence of nodulation, with the criterion for categorising particle size and colour detailed in S2 Table, and raw data presented in S4 Table. Multiple individual guanos from each sample were assessed using callipers in order to define maximum and minimum measurements for each sample. Nodulation was observed by eye. In order to ensure consistency between measurements, all of the measurements were conducted under the supervision of one researcher.

### Guano species identification

To ensure correct species identification, guano samples were identified by DNA barcoding. Individual guano samples were crushed and incubated overnight at 37°C in 300 μl CTAB buffer on a sample agitator at 400rpm. After incubation, DNA was extracted using chloroform:isoamyl alcohol 24:1. After spinning, the DNA is in the aqueous phase, and proteins and polysaccharides move into the chloroform/alcohol layer, removing these inhibitors. The DNA was then purified using DNeasy columns and buffers, with an additional acetone wash and dry before elution [28, 29].

The species of bat was confirmed using barcoding as follows; 20 μl PCRs were prepared using a mixture of all of the primers shown in S5 Table, each at 5 μM. Each PCR contained 2 μl 10X Platinum® Taq buffer, 2 μl of dNTPs at 2mM, 0.8μl 50mM Mg2+, 1.3 μl primer mix, 0.1 μl Platinum® Taq DNA polymerase, between 0.2–2 μl of sample and 11.8–13.6 μl ultrapure H_2_O. Touchdown PCR was used in order to account for the differences in optimum annealing temperatures of the primers used [30, 31]. Touchdown thermal cycling conditions were as follows: 5 mins at 95°C, followed by 10 cycles of 94°C for 30s, 57°C for 30s (decreasing by 0.1°C per cycle) and 72°C for 30s, followed by 32 cycles of 95°C for 30s, 54°C for 30s, then 72°C for 30s, followed by a final extension period of 72°C for 7 minutes.

After PCR, success was determined by running on a 2% agarose gel, stained with gel red. Clean-up was undertaken by adding 2 μl of Fast-AP and 0.5 μl of Exonuclease-1, then incubated at 37°C for 30mins, then 80°C for 15 mins. Forward primers (BF1-7) were used in a GATC Lightrun^TM^ Sanger sequencing reaction. Sequences were checked from traces using CodonCode aligner, then sequences were confirmed using the NCBI database.

This process resulted in a sample size of 104 positively identified samples from the 16 target species to compare with the diet dataset. A summary of sample sizes per species for both the diet and guano datasets is available in S6 Table.

### Statistical analysis

**Fig 1:**
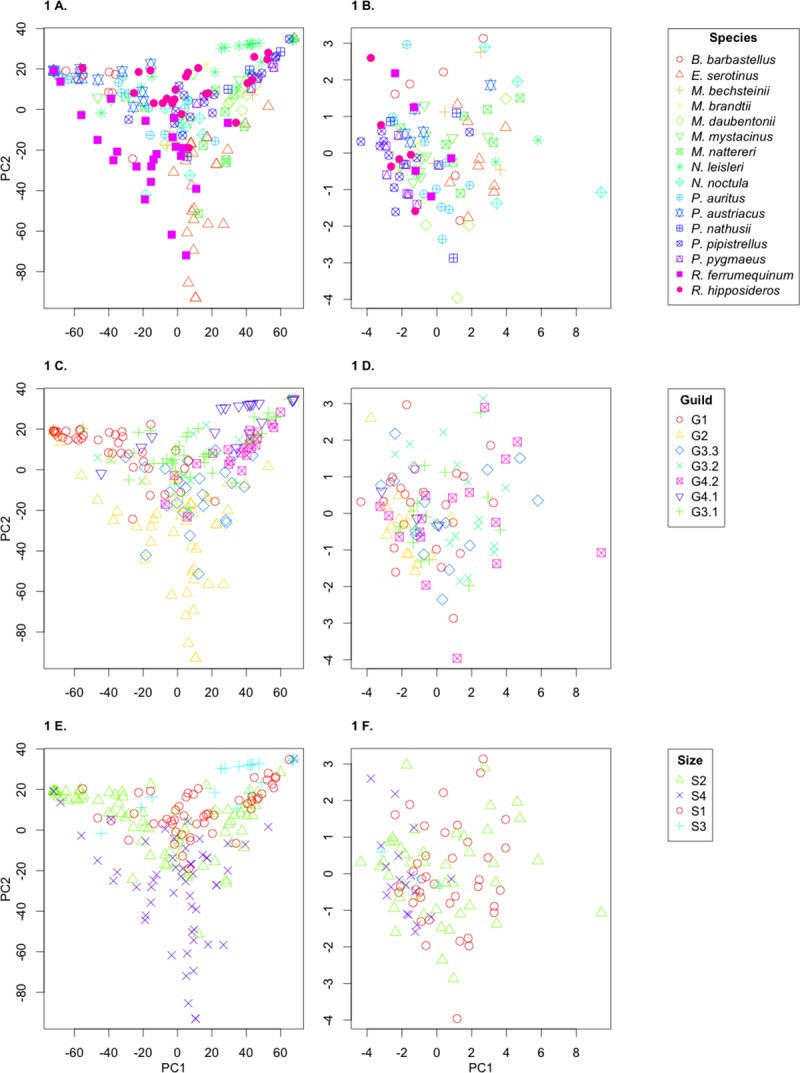
a-f. Principle component analysis plots of both diet (A, C, and E) and guano (B, D, and F) data. Data assigned by species (A,B), guild (C,D) and size (E,F).

In order to compare between diet and guano datasets, Principle Component Analysis (PCA) was undertaken using R function prcomp using default settings [32]. PCA plots comparing principle component 1 (PC1) and principle component 2 (PC2) for the diet dataset and the guano dataset were created using R and data was coloured by 1) species, 2) dietary guild and 3) size of bat. To assign dietary guilds, diets as determined from the literature (S2 Table, Fig 1) were sorted using complete-linkage clustering using the hclust package in R, to group diets by the average composition for each species. This allowed us to delimit the data into 7 dietary guilds (*M*. *alcathoe* was excluded from later analysis, so was not assigned to a guild. To assign size categories, the minimum and maximum weight for each bat species were gathered from the Bat Conservation Trust (http://www.bats.org.uk/pages/uk_bats.html#Resident) and plotted to visualise size groupings (please see S1 Fig).

Spatial autocorrelation within PC1 and PC2 for diet and guano respectively was calculated using the Moran.I function in the ape v5.3 R package with default settings [33] to assess whether the clustering of the assigned groups was significant.

Wilcoxon signed-rank test was conducted comparing PC1 for diet to PC1 for guano, PC2 for diet against PC2 for guano etc. This allowed us to use the individual sample dataset retaining the variation within each grouping and look at one proposed cluster at a time. This method was used to compare clusters assigned by 1) species, 2) dietary guild and 3) size.

## Results

Fig 1 shows PCA plots showing clusters assigned to species for diet (Fig 1A, 1C and 1E) and guano (Fig 1B, 1D and 1F) respectively. PC1 represents 58.24% and 54.83% of the proportion of variance for diet and guano. With PC1 and PC2 cumulatively representing 83.39% and 70.46% of the data for diet and guano, respectively. For diet, the Moran’s I analysis when the data is assigned to species shows significant clustering (*P* = 2.510526e-05), this was expected because the diet data carries the assumption that the same species will have a similar diet, therefore this result is a product of the method. Clustering the guano data by species also yielded a significant result (*P* = 3.352874e-14). This was investigated further with Wilcoxen signed-rank test comparison of PC1, which represents the highest proportion of variance for both datasets. This analysis revealed 9 species out of the 16 had a significant correlation between diet and guano morphology in PC1 (*B*. *barbastellus*, *E*. *serotinus*, *M*. *daubentonii*, *M*. *nattereri*, *P*. *auritus*, *P*. *austriacus*, *P*. *nathusii*, *P*. *pipistrellus*, and *P*. *pygmaeus*) (see S7A Table.

**Fig 2:**
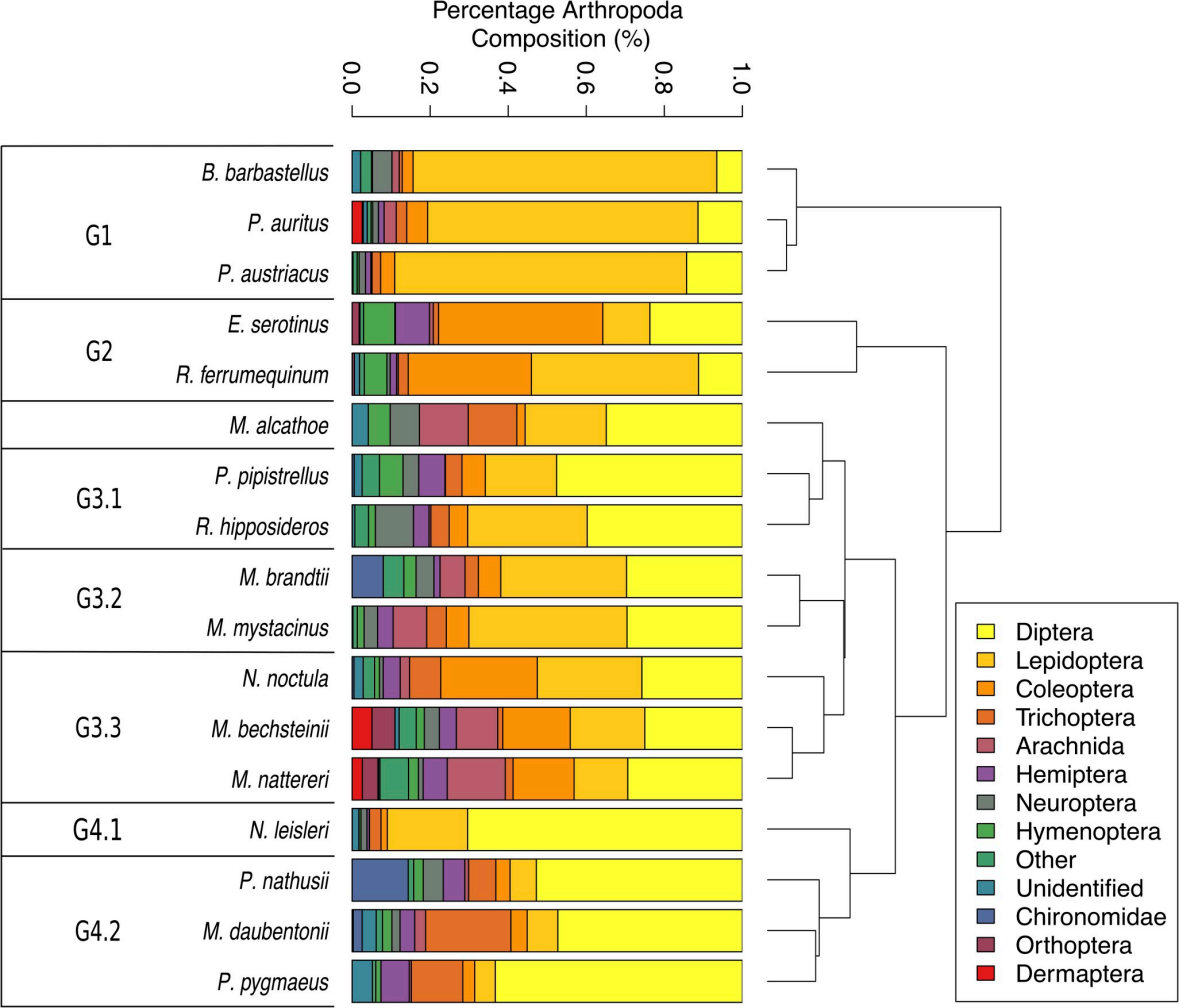
The diet of each bat species as identified from the literature review. The data presented is the mean diet of each of the bat species (for raw data, see S2 Table). Diets sorted using complete-linkage clustering using hclust in R.

The data was then grouped into dietary guilds (Fig 2) with guild 1 (G1) composed of bat species with a diet of largely Lepidoptera; Guild 2, bat species with diets containing largely Coleoptera; Guild 3, which was identified using the complete-linkage clustering analysis, was broken down further into G3.1, generalist and Diptera diet; G3.2, generalist, Lepidoptera and Diptera diet and G3.3, generalist with Coleoptera. Guild 4 from complete-linkage clustering was broken down to G4.1, a diet consisting of almost all Diptera, and G4.2 a diet consisting of largely Diptera with notable Trichoptera presence.

Fig 1C and 1D, show PCA plots assigned to dietary guilds (defined in Fig 2) for diet and guano respectively. The clustering of guilds within the diet data is highly significant (*P* = <0.001) which is exactly as expected because the guilds were assigned using the diet data. However, interestingly the guilds also produce significant clustering within the guano data (*P* = 0.0156828). Wilcoxen’s signed rank test comparison revealed this was mainly driven by G1, G3.1, and G4.2 (please see S7B Table).

Fig 1E and 1F show PCA plots assigned to bat size (defined in S1 Fig) for diet (Fig 1E) and guano (Fig 1F). Moran’s I analysis shows the clustering for both diet and guano was highly significant (*P* = <0.001) when assigned by size, somewhat matching the distributions within dietary guild. Wilcoxen’s signed rank test comparison revealed all four size groupings were significantly correlating when comparing PC2 v PC2 but only Size category S1, the smallest bat species were significant when comparing PC1 of both data sets (please see S7B Table).

## Discussion

The aim of this study was to identify whether bat guano morphology data could be used to predict bat diet. We first compared bat diet and bat guano morphology across all the species of British bat. As expected, the guano morphology overlapped too extensively to be able to distinguish between each bat species. It is nevertheless interesting that nine species when investigated individually do seem to correlate between diet and guano morphology. Fig 1B shows it is possible to distinguish a few species from each other for example, *P*. *pipistrellus* rarely overlaps with *E*. *serotinus*. Generally, however, it would be impossible to distinguish between for example *M*. *daubentonii*, *P*. *austriacus*, *P*. *nathusii*, and *P*. *auritus* as they all overlap considerably in terms of guano morphology.

However, grouping the data by dietary guilds shows significant clusters within the guild morphology data. Though the clusters still overlap there is a pattern within the data particularly between Guild 1 (diet consisting of largely Lepidoptera), Guild 3.1, (generalist and Diptera diet), Guild 3.3 (generalist with Coleoptera) and Guild 4.2, (largely Diptera with notable Trichoptera presence), where species following these diets generally produce similar size and shape of guano. When looking at grouping by the size of the bat, there is a similar pattern to that shown in dietary guild, with the largest separation between the smallest (S1) species and the largest (S4) species. Considering that the size and diet of a bat is intrinsically linked with one another [34] it would be hard to pull apart their separate influences on the guano morphology data. A species within the smallest group of bats is more likely to have a very different diet than a species in the largest group of bats, for example, *P*. *pipistrellus* and *R*. *hipposideros* are both in S1 and G3.1 and are quite different to *E*. *serotinus* and *R*. *ferrumequinum* who are both in S4 and G2, therefore it is unknown the true cause of the divergence.

It has previously been observed that bats which feed primarily on Coleoptera and Diptera tend to have longer, more robust guano than those feeding on Lepidoptera [27]. Here, we also find that the three largest bats (*E*. *serotinus*, *R*. *ferrumequinum*, and *N*. *noctula*) do have the largest proportions of Coleoptera in their diets (Fig 2), and typically have large particle sizes, despite not always having the largest guano (S4 Table). In particular, we found that despite being amongst the largest bats, *R*. *ferrumequinum* typically has considerably shorter guano than previously observed; in our study the length range was 2.5–9.5mm (in comparison to a length of 9-13mm previously reported [35]). We do not see any clear relationship with bat size for those selecting Lepidoptera or Diptera, although some of the smallest bats (i.e. *P*. *pygmaeus*) have high proportions of their diets comprised of Diptera, in contrast to previous observations [35]. In *B*. *barbastellus*, which has the largest proportion of Lepidoptera found within the diet, we observe that the guanos were the lightest in colour, with moderate particle sizes (S4 Table). Nodulation (S4 Table) can occur in any species, although many species have both nodulated and non-nodulated guano observed. In general, guano morphology seems more correlated to diet than species. This enables the identification of the most important prey taxa within a local environment; a crucial step for informing conservation strategies.

## Supporting information

S1 Table Method of diet analysis of diets retrieved from the literature. The column labelled “Code” is used in S2 Table.
(DOCX)

S2 Table Diet data as retrieved from the literature. Including information pertaining to the species studied, the study reference, the location in which the study took place, the number of individuals sampled, the study method (See S1 Table), and the diet data. See S2 Table.
(CSV)

S3 Table Criterion used in categorizing guano particle size (a) and colour (b).
(DOCX)

S4 Table Primers used to confirm the identity of the bat species which produced the guano. Forward and reverse primers shown in a) and b) respectively.
(CSV)

S5 Table Sample sizes for a) each species, b) each dietary guild and c) each size category.
(DOCX)

S6 Table Measurements of the guano taken. See S4 Table.
(DOCX)

S7 Table The results of Wilcoxen signed rank test comparing the PCA outputs of Diet and Guano morphology. Only significant correlations are presented: * *P*-value = <0.05, ** *P*-value = <0.01, *** *P*-value = <0.001.
(DOCX)

S1 Fig The division British bat species into size categories defined by minimum and maximum weight. Weights gathered for each species from the Bat Conservation Trust website (http://www.bats.org.uk/pages/uk_bats.html#Resident).
(DOCX)

S2 Fig Dietary diversity and niche breadth of each species. The dietary diversity calculated using Shannon-Weaver diversity index (H’) (grey) and niche breadth calculated using Levin’s standardised index (B_A_) (black).
(DOCX)

S1 File (DOCX)
