# Supplementary material for: Guano morphology has the potential to inform conservation strategies in British bats
Source: PLoS One. 2020 Apr 9;15(4):e0230865. doi: 10.1371/journal.pone.0230865 (PMC7145103; doi:10.1371/journal.pone.0230865)
Supplement: S1 Table — The column labelled “Code” is used in S2 Table. (DOCX) [file pone.0230865.s001.docx]

**S1 Table. Method of diet analysis of diets retrieved from the literature.** The column labelled “Code” is used in S2 Table.

| **Code** | **Method of diet analysis** |
| --- | --- |
| A | Percentage volume from stomach contents |
| Bi | Percentage volume from faecal pellets |
| Bii | Percentage occurrence from faecal pellets |
| Biii | Percentage frequency from faecal pellets |
| Biv | Percentage items (Vaughan, 1997) from faecal pellets |
| Bv | Percentage numbers (Vaughan, 1997) from faecal pellets |
| Bvi | Percentage animals |
| C | Frequency of items from stomach |
| D | Frequency of prey remains collected from below feeding perches |
| E | Molecular |
| F | Anecdotal/ direct observation |
| G | Isotope analysis |
| ? | Unknown |
