# Supplementary material for: Guano morphology has the potential to inform conservation strategies in British bats
Source: PLoS One. 2020 Apr 9;15(4):e0230865. doi: 10.1371/journal.pone.0230865 (PMC7145103; doi:10.1371/journal.pone.0230865)
Supplement: S3 Table — Criterion used in categorizing guano particle size (a) and colour (b). (DOCX) [file pone.0230865.s003.docx]

**S3 Table.** **Criterion used in categorizing guano particle size (a) and colour (b)**

1. Particle size

| 1. | Very fine, smooth outline, small divots |
| --- | --- |
| 2. | Fine, mostly smooth outline, bigger divots |
| 3. | Medium size, rough outline, medium divots |
| 4. | Quite coarse, rough outline, medium to large divots |
| 5. | Coarse, very rough outline, large divots |

1. Colour

| 1. | Light to dark yellow |
| --- | --- |
| 2. | Light brown with yellow flecks present |
| 3. | Light-medium brown |
| 4. | Medium-dark brown with some black flecks |
| 5. | Over half of the guano is black |
