# Supplementary material for: Guano morphology has the potential to inform conservation strategies in British bats
Source: PLoS One. 2020 Apr 9;15(4):e0230865. doi: 10.1371/journal.pone.0230865 (PMC7145103; doi:10.1371/journal.pone.0230865)
Supplement: S5 Table — Sample sizes for a) each species, b) each dietary guild and c) each size category. (DOCX) [file pone.0230865.s005.docx]

**S5 Table.** **Primers used to confirm the identity of the bat species which produced the guano.**

1. Forward primers

| **Primer name** | **Orientation** | **Sequence** |
| --- | --- | --- |
| BF1 | Forward | ATGACAAACAYTCGAAAATCC |
| BF2 | Forward | ATGACAAACATTCGAAAGTMC |
| BF3 | Forward | ATGACCAACATTCGTAAATCW |
| BF4 | Forward | ATGACCAACATTCGAAAATCY |
| BF5 | Forward | ATGACCMACATTCGAAAATCY |
| BF6 | Forward | ATGACCAACATTCGAAAGTCY |
| BF7 | Forward | ATGACCAACATTCGCAARTCY |
| BX1 | Reverse | GTCTGMTGTRTAGTGTATGG |

1. Reverse primers

| BX2 | Reverse | RTCYGATGTGTGATGCATGG |
| --- | --- | --- |
| BX3 | Reverse | RTCTGATGTRTAGTGTATTGC |
| BX4 | Reverse | RTCTGATGTRTARTGTATGGC |
| BX5 | Reverse | RTCTGAYGTRTAGTGTATAGC |
| BX6 | Reverse | RTCTGATRTGTAATGTATAGC |
| BX7 | Reverse | ATCTGATGTATAATGTATWGCT |
| BX8 | Reverse | GTCTGATGTATAGTGTATGGA |
| BX9 | Reverse | GTCTGGTGTGTAATGTATGG |
| BX10 | Reverse | ATCTGATGTAGTGCGCATGG |
