# Supplementary material for: Guano morphology has the potential to inform conservation strategies in British bats
Source: PLoS One. 2020 Apr 9;15(4):e0230865. doi: 10.1371/journal.pone.0230865 (PMC7145103; doi:10.1371/journal.pone.0230865)
Supplement: S6 Table — See S4 Table. (DOCX) [file pone.0230865.s006.docx]

**S6 Table.** **Sample sizes for each a) species, b) dietary guild, and c) size category.** Guano sample size is the number of guano assemblages measured (S4 Table), diet sample size is the number of diets identified from the literature (S2 Table).

1. Species

| **Species** | **Guano sample size** | **Diet sample size** |
| --- | --- | --- |
| *Barbastellus barbastellus* | 6 | 11 |
| *Eptesicus serotinus* | 9 | 26 |
| *Myotis bechsteinii* | 4 | 5 |
| *Myotis brandtii* | 4 | 3 |
| *Myotis daubentonii* | 5 | 13 |
| *Myotis mystacinus* | 11 | 5 |
| *Myotis nattereri* | 8 | 13 |
| *Nyctalus leisleri* | 3 | 19 |
| *Nyctalus noctula* | 4 | 7 |
| *Plecotus auritus* | 12 | 26 |
| *Plecotus austriacus* | 5 | 11 |
| *Pipistrellus nathusii* | 4 | 4 |
| *Pipistrellus pipistrellus* | 10 | 16 |
| *Pipistrellus pygmaeus* | 8 | 5 |
| *Rhinolophus ferrumequinum* | 5 | 23 |
| *Rhinolophus hipposideros* | 6 | 24 |
| *Myotis alcathoe* | - | 4 |
| **Total** | **104** | **215** (211 without *M. alcathoe*) |

1. Guild

| **Guild** | **Diet** | **Guano** |
| --- | --- | --- |
| G1 | 48 | 23 |
| G2 | 49 | 14 |
| G3.1 | 41 | 16 |
| G3.2 | 8 | 15 |
| G3.3 | 25 | 16 |
| G4.1 | 19 | 3 |
| G4.2 | 22 | 17 |

1. Size

| **Size class** | **Diet** | **Guano** |
| --- | --- | --- |
| S1 | 51 | 39 |
| S2 | 86 | 44 |
| S3 | 19 | 3 |
| S4 | 56 | 18 |
