# Supplementary material for: Guano morphology has the potential to inform conservation strategies in British bats
Source: PLoS One. 2020 Apr 9;15(4):e0230865. doi: 10.1371/journal.pone.0230865 (PMC7145103; doi:10.1371/journal.pone.0230865)
Supplement: S7 Table — Only significant correlations are presented: * P-value = <0.05, ** P-value = <0.01, *** P-value = <0.001. (DOCX) [file pone.0230865.s007.docx]

**S7 Table.** **The results of Wilcoxen signed rank test comparing the PCA outputs of Diet and Guano morphology.**

1. Species

| **Species** | **Component 1** | **Component 2** | **Component 3** | **Component 4** |
| --- | --- | --- | --- | --- |
| Diet data: proportion of variance | 58.24% | 25.15% | 5.608% | 2.299% |
| Guano data: proportion of variance | 53.83% | 15.63% | 13.59% | 11.56% |
| *B. barbastellus* | *** | *** |  |  |
| *E. serotinus* | *** | *** |  |  |
| *M. bechsteinii* |  |  |  |  |
| *M. brandtii* |  |  |  |  |
| *M. daubentonii* | * |  | *** | ** |
| *M. mystacinus* |  |  |  |  |
| *M. nattereri* | * |  |  | ** |
| *N. leisleri* |  | ** |  |  |
| *N. noctula* |  |  |  |  |
| *P. auritus* | ** | ** |  |  |
| *P. austriacus* | ** | ** | * |  |
| *P. nathusii* | * |  |  |  |
| *P. pipistrellus* | *** |  |  |  |
| *P. pygmaeus* | ** | ** |  |  |
| *R. ferrumequinum* |  | ** |  |  |
| *R. hipposideros* |  | ** |  |  |

1. Guild

| **Guild** | **Component 1** | **Component 2** | **Component 3** | **Component 4** |
| --- | --- | --- | --- | --- |
| G1 | *** | *** | * |  |
| G2 |  | *** | * |  |
| G3.1 | ** | *** |  |  |
| G3.2 |  |  |  |  |
| G3.3 |  | * |  | * |
| G4.1 |  | ** |  |  |
| G4.2 | *** | *** | *** | ** |

1. Size

| **Size** | **Component 1** | **Component 2** | **Component 3** | **Component 4** |
| --- | --- | --- | --- | --- |
| S1 | *** | *** |  |  |
| S2 |  | *** |  |  |
| S3 |  | ** |  |  |
| S4 |  | *** | ** |  |

Only significant correlations are presented

* p-value = <0.05, ** p-value = <0.01, *** p-value = <0.001.
