# Supplementary material for: Guano morphology has the potential to inform conservation strategies in British bats
Source: PLoS One. 2020 Apr 9;15(4):e0230865. doi: 10.1371/journal.pone.0230865 (PMC7145103; doi:10.1371/journal.pone.0230865)
Supplement: S1 Fig — Weights gathered for each species from the Bat Conservation Trust website (http://www.bats.org.uk/pages/uk_bats.html#Resident). (DOCX) [file pone.0230865.s008.docx]

S1 S2 S3 S4

**S1 Fig.** **The division British bat species into size categories defined by minimum and maximum weight.** Weights gathered for each species from the Bat Conservation Trust website (http://www.bats.org.uk/pages/uk_bats.html#Resident)
