# Supplementary material for: Guano morphology has the potential to inform conservation strategies in British bats
Source: PLoS One. 2020 Apr 9;15(4):e0230865. doi: 10.1371/journal.pone.0230865 (PMC7145103; doi:10.1371/journal.pone.0230865)
Supplement: S2 Fig — The dietary diversity calculated using Shannon-Weaver diversity index (H’) (grey) and niche breadth calculated using Levin’s standardised index (BA) (black). (DOCX) [file pone.0230865.s009.docx]

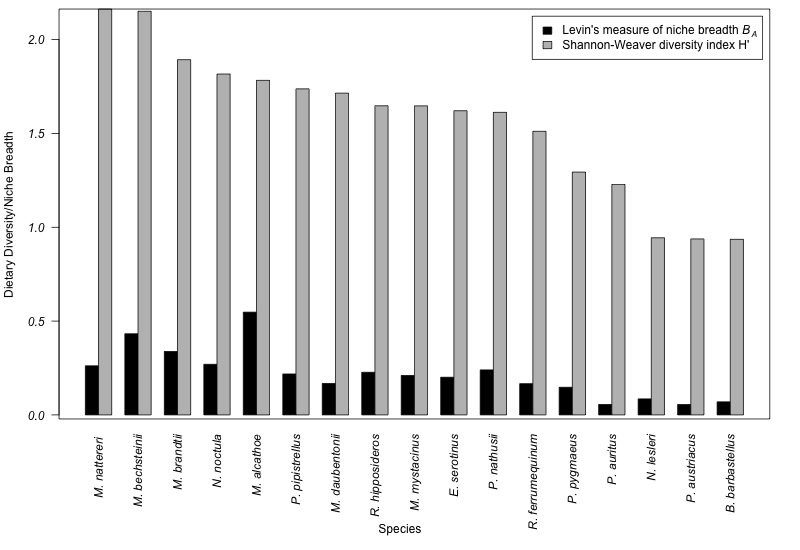


**Figure. S2.** Dietary diversity and niche breadth of each species. The dietary diversity calculated using Shannon-Weaver diversity index (H’) (grey) and niche breadth calculated using Levin’s standardised index (B_A_) (black).
