# Supplementary material for: Guano morphology has the potential to inform conservation strategies in British bats
Source: PLoS One. 2020 Apr 9;15(4):e0230865. doi: 10.1371/journal.pone.0230865 (PMC7145103; doi:10.1371/journal.pone.0230865)
Supplement: S1 File — (DOCX) [file pone.0230865.s010.docx]

# **Supplementary references**

1. Ahmim M, Moali A. The diet of four species of horseshoe bat (Chiroptera: Rhinolophidae) in a mountainous region of Algeria: evidence for gleaning. Hystrix, the Italian Journal of Mammalogy. 2013;24(2):4.

2. Andreas M. Study on bat diet in the Czech Republic and Slovakia. A Tribute to Bats, Prague. 2010:261-4.

3. Andreas M, Reiter A, Cepáková E, Uhrin M. Body size as an important factor determining trophic niche partitioning in three syntopic rhinolophid bat species. Biologia. 2013;68(1):170-5.

4. Andreas M, Reiter A, Benda P. Prey selection and seasonal diet changes in the western barbastelle bat (*Barbastella barbastellus*). Acta Chiropterologica. 2012;14(1):81-92.

5. Andreas M, Reiter A, Benda P. Dietary composition, resource partitioning and trophic niche overlap in three forest foliage-gleaning bats in Central Europe. Acta Chiropterologica. 2012;14(2):335-45.

6. Arlettaz R, Godat S, Meyer H. Competition for food by expanding pipistrelle bat populations (*Pipistrellus pipistrellus*) might contribute to the decline of lesser horseshoe bats (*Rhinolophus hipposideros*). Biological Conservation. 2000;93(1):55-60.

7. Ashrafi S, Beck A, Rutishauser M, Arlettaz R, Bontadina F. Trophic niche partitioning of cryptic species of long-eared bats in Switzerland: implications for conservation. European Journal of Wildlife Research. 2011;57(4):843-9.

8. Barlow KE. The diets of two phonic types of the bat *Pipistrellus pipistrellus* in Britain. Journal of Zoology. 1997;243(3):597-609.

9. Bárta Z. K potravě netopýra ušatého (*Plecotus auritus* L.). Lynx, n s. 1975;17:5-6.

10. Bartonička T, Řehák Z, Andreas M. Diet composition and foraging activity of *Pipistrellus pygmaeus* in a floodplain forest. Biologia. 2008;63(2):266-72.

11. Bauerova Z. Contribution to the trophic ecology of the grey long-eared bat, *Plecotus austriacus*. Folia Zoologica. 1982;31:113-22.

12. Bauerová Z, Cerveny J. Towards an understanding of the trophic ecology of *Myotis nattereri*. Folia Zoologica. 1986;35(1):55-61.

13. Beck A. Fecal analyses of European bat species. Myotis. 1995;32(33):109-19.

14. Beck A, Bontadina F, Gloor S, Hotz T, Lutz M, Muehlethaler E. Jagdhabitatwahl und nächtliche Aufenthaltsgebiete der Grossen Hufeisennase (*Rhinolophus ferrumequinum*) im Raum Castrich/GR. Arbeitsgruppe zum Schutz der Hufeisennasen Graubündens ASGH, Encarden. 1994;51:7152.

15. Beck A. Nahrungsuntersuchungen bei der Fransenfledermaus, *Myotis nattereri* (Kuhl, 1818). Myotis. 1991;29:67-70.

16. Beck A, Stutz H, Ziswiler V. Nutritional habits of the lesser horseshoe bat *Rhinolophus hipposideros* (Bechstein, 1800)(Mammalia, Chiroptera). Revue Suisse de Zoologie. 1989;96(3):643-50.

17. Bontadina F, Schmied SF, Beck A, Arlettaz R. Changes in prey abundance unlikely to explain the demography of a critically endangered Central European bat. Journal of Applied Ecology. 2008;45(2):641-8.

18. Boonman M. Voedselkeuze grootoorvleermuis. jrg. 6(1995)nr. 2 ed: Zoogdier; 1995.

19. Buckhurst A. Moths destroyed by a long eared bat. Entomologist. 1930;63:238.

20. Catto C, Hutson A, Racey P. The diet of *Eptesicus serotinus* in southern England. Folia Zoologica. 1994;43(4):307-14.

21. Chung C-U, Han S-H, Cha J-Y, Kim S-C, Kim J-J, Jeong J-C, et al. The diet composition of the serotine bat, *Eptesicus serotinus* revealed by faecal analysis. Korean Journal of Environmental Ecology. 2015;29(3):368-73.

22. Danko Š, Krištín A, Krištofík J. *Myotis alcathoe* in eastern Slovakia: Occurrence, diet, ectoparasites and notes on its identification in the field. Vespertilio. 2010;13:77-91.

23. Vaughan N. The diets of British bats (Chiroptera). Mammal Review. 1997;27(2):77-94.

24. Feldman R, Whitaker JO, Yom-Tov Y. Dietary composition and habitat use in a desert insectivorous bat community in Israel. Acta Chiropterologica. 2000;2(1):15-22.

25. Flanders J, Jones G. Roost use, ranging behavior, and diet of greater horseshoe bats (*Rhinolophus ferrumequinum*) using a transitional roost. Journal of Mammalogy. 2009;90(4):888-96.

26. Flavin DA, Biggane SS, Shiel CB, Smiddy P, Fairley JS. Analysis of the diet of Daubenton’s bat *Myotis daubentonii* in Ireland. Acta Theriologica. 2001;46(1):43-52.

27. Gajdosik M, Gaisler J. Diet of two Eptesicus bat species in Moravia (Czech Republic). Folia Zoologica. 2004;53(1):7-16.

28. Gerber E, Haffner M, Ziswiler V. Comparative food analysis of the serotine bat *Eptesinus serotinus* (Schreber, 1774) (Mammalia, Chiroptera) in different regions of Switzerland. Revue Suisse de Zoologie. 1994;101(4):858.

29. Gloor S, Stutz H-PB, Ziswiler V. Nutritional habits of the noctule bat *Nyctalus noctula* (Schreber, 1714) in Switzerland. Myotis. 1989;32:231-42.

30. Heinicke W, Krauß A. Zum beutespektrum des braunen langohrs, *Plecotus auritus*. Nyctalus (NF). 1978;1:49-52.

31. Hoare L. The diet of *Pipistrellus pipistrellus* during the pre‐hibernal period. Journal of Zoology. 1991;225(4):665-70.

32. Hope PR, Bohmann K, Gilbert MTP, Zepeda-Mendoza ML, Razgour O, Jones G. Second generation sequencing and morphological faecal analysis reveal unexpected foraging behaviour by *Myotis nattereri* (Chiroptera, Vespertilionidae) in winter. Frontiers in Zoology. 2014;11(1):39.

33. Jin L, Feng J, Sun K, Liu Y, Wu L, Li Z, et al. Foraging strategies in the greater horseshoe bat (*Rhinolophus ferrumequinum*) on Lepidoptera in summer. Chinese Science Bulletin. 2005;50(14):1477-82.

34. Wang Jing WX, Jiang Tinglei,Wang Lei,Lu Guanjun,You Yuyan,Liu Ying,Li Dan,Feng Jiang. Relationships between foraging activity of greater horseshoe bat (*Rhinolophus ferrumequinum*) and prey resources. Acta Theriologica Sinica. 2010;30(2):157-62.

35. Jones G. Flight performance, echolocation and foraging behaviour in noctule bats *Nyctalus noctula*. Journal of Zoology. 1995;237(2):303-12.

36. Jones G. Prey selection by the greater horseshoe bat (*Rhinolophus ferrumequinum*): optimal foraging by echolocation? The Journal of Animal Ecology. 1990:587-602.

37. Kaňuch P, Janečková K, Krištín A. Winter diet of the noctule bat *Nyctalus noctula*. Folia Zoologica. 2005;54(1-2):53-60.

38. Kaňuch P, Krištín A, Krištofík J. Phenology, diet, and ectoparasites of Leisler's bat (*Nyctalus leisleri*) in the Western Carpathians (Slovakia). Acta Chiropterologica. 2005;7(2):249-57.

39. Kervyn T, Libois R. The diet of the serotine bat: A comparison between rural and urban environments. Belgian Journal of Zoology. 2008;138(1).

40. Krauss A. Materials on foodstuff biology of long-eared bat *Plecotus auritus*, Mammalia, Chiroptera. Zoologische Abhandlungen (Dresden). 1978;34(22):325-38.

41. Krüger F, Clare E, Greif S, Siemers B, Symondson W, Sommer R. An integrative approach to detect subtle trophic niche differentiation in the sympatric trawling bat species *Myotis dasycneme* and *Myotis daubentonii*. Molecular Ecology. 2013;23(15):3657-71.

42. Krüger F, Clare EL, Symondson WO, Keišs O, Pētersons G. Diet of the insectivorous bat *Pipistrellus nathusii* during autumn migration and summer residence. Molecular Ecology. 2013;23(15):3672-83.

43. Krüger F, Harms I, Fichtner A, Wolz I, Sommer RS. High trophic similarity in the sympatric North European trawling bat species *Myotis daubentonii* and *Myotis dasycneme*. Acta Chiropterologica. 2012;14(2):347-56.

44. Lino A, Fonseca C, Goiti U, Pereira MJR. Prey selection by *Rhinolophus hipposideros* (Chiroptera, Rhinolophidae) in a modified forest in Southwest Europe. Acta Chiropterologica. 2014;16(1):75-83.

45. Lucan RK, Andreas M, Benda P, Bartonicka T, Brezinová T, Hoffmannová A, et al. Alcathoe bat (*Myotis alcathoe*) in the Czech Republic: distributional status, roosting and feeding ecology. Acta Chiropterologica. 2009;11(1):61-9.

46. Ma J, Liang B, Zhang S, Metzner W. Dietary composition and echolocation call design of three sympatric insectivorous bat species from China. Ecological Research. 2008;23(1):113-9.

47. Mackenzie G, Oxford G. Prey of the noctule bat (*Nyctalus noctula*) in East Yorkshire. Journal of Zoology. 1995;236(2):322-7.

48. Manwaring J. Lepidoptera taken by bats. Entomologist. 1939;72:190.

49. McAney C, Fairley J. Analysis of the diet of the lesser horseshoe bat *Rhinolophus hipposideros* in the West of Ireland. Journal of Zoology. 1989;217(3):491-8.

50. McAney C. The analysis of bat droppings: Mammal Society; 1991.

51. Mikula P, Čmoková A. Lepidopterans in the summer diet of *Eptesicus serotinus* in Central Bohemia. Vespertilio. 2012;16:197–201.

52. Nissen H, Krueger F, Fichtner A, Sommer RS. Local variability in the diet of Daubenton's bat (*Myotis daubentonii*) in a lake landscape of northern Germany. Folia Zoologica. 2013;62(1):36-41.

53. Poulton EB. British insectivorous bats and their prey. Proceedings of the Zoological Society of London. 1929;99(2):277-303.

54. Razgour O, Clare EL, Zeale MRK, Hanmer J, Schnell IB, Rasmussen M, et al. High‐throughput sequencing offers insight into mechanisms of resource partitioning in cryptic bat species. Ecology and Evolution. 2011;1(4):556-70.

55. Robinson M. Prey selection by the brown long-eared bat (*Plecotus auritus*). Myotis. 1990;28:5-18.

56. Robinson M, Stebbings R. Food of the serotine bat, *Eptesicus serotinus*—is faecal analysis a valid qualitative and quantitative technique? Journal of Zoology. 1993;231(2):239-48.

57. Rostovskaya MS, Zhukova DV, Illarionova AE, Ustyugova SV, Borissenko AV, Sviridov AV. Insect prey of the Long-eared bat *Plecotus auritus* (L.)(Chiroptera: Vespertilionidae) in Central Russia. Russian Entomological Journal. 2000;9:185-9.

58. Roswag A, Becker NI, Encarnação JA. Importance of multi-dimensional analyses of resource partitioning in highly mobile species assemblages. Population Ecology. 2015;57(4):601-11.

59. Rydell J. Food habits of northern (*Eptesicus nilssoni*) and brown long‐eared (*Plecotus auritus*) bats in Sweden. Ecography. 1989;12(1):16-20.

60. Rydell J, Natuschke G, Theiler A, Zingg PE. Food habits of the barbastelle bat *Barbastella barbastellus*. Ecography. 1996;19(1):62-6.

61. Siemers BM, Swift SM. Differences in sensory ecology contribute to resource partitioning in the bats *Myotis bechstein*ii and *Myotis nattereri* (Chiroptera: Vespertilionidae). Behavioral Ecology and Sociobiology. 2006;59(3):373-80.

62. Shiel C, McAney C, Fairley J. Analysis of the diet of Natterer's bat *Myotis nattereri* and the common long‐eared bat *Plecotus auritus* in the west of Ireland. Journal of Zoology. 1991;223(2):299-305.

63. Shiel CB, Duverge PL, Smiddy P, Fairley JS. Analysis of the diet of Leisler's bat (*Nyctalus leisleri*) in Ireland with some comparative analyses from England and Germany. Journal of Zoology. 1998;246(04):417-25.

64. Sierro A, Arlettaz R. Barbastelle bats (*Barbastella spp*.) specialize in the predation of moths: implications for foraging tactics and conservation. Acta Oecologica. 1997;18(2):91-106.

65. Smirnov D, Vekhnik V. Ecology of nutrition and differentiation of the trophic niches of bats (Chiroptera: Vespertilionidae) in floodplain ecosystems of the Samara Bend. Biology Bulletin. 2014;41(1):60-70.

66. Sologor EA. Kizuchenyu pitaniya *Vespertilio serotinus*. Voprosy Teriologii Rukokrylye. 1980:188-90.

67. Sullivan C, Shiel C, McAney C, Fairley J. Analysis of the diets of Leisler's *Nyctalus leisleri,* Daubenton's *Myotis daubentoni* and Pipistrelle *Pipistrellus pipistrellus* bats in Ireland. Journal of Zoology. 1993;231(4):656-63.

68. Swift S, Racey P. Resource partitioning in two species of vespertilionid bats (Chiroptera) occupying the same roost. Journal of Zoology. 1983;200(2):249-59.

69. Swift S, Racey P, Avery M. Feeding ecology of *Pipistrellus pipistrellus* (Chiroptera: Vespertilionidae) during pregnancy and lactation. II. Diet. The Journal of Animal Ecology. 1985;54:217-25.

70. Taake K. Zur Nahrungsökologie waldbewohnender Fledermäuse (Chiroptera, Vespertilionidae)—ein Nachtrag. Myotis. 1993;31:163-5.

71. Thompson M. A common long-eared bat *Plecotus auritus*: moth predator-prey relationship. Naturalist. 1982;107:87-97.

72. Vesterinen EJ, Lilley T, Laine VN, Wahlberg N. Next generation sequencing of fecal DNA reveals the dietary diversity of the widespread insectivorous predator Daubenton’s Bat (*Myotis daubentonii*) in Southwestern Finland. PLoS ONE. 2013;8(11):e82168.

73. Waters D, Jones G, Furlong M. Foraging ecology of Leisler's bat (*Nyctalus leisleri*) at two sites in southern Britain. Journal of Zoology. 1999;249(2):173-80.

74. Waters DA, Rydell J, Jones G. Echolocation call design and limits on prey size: a case study using the aerial-hawking bat *Nyctalus leisleri*. Behavioral Ecology and Sociobiology. 1995;37(5):321-8.

75. Whitaker Jr JO, Karatas A. Food and feeding habits of some bats from Turkey. Acta Chiropterologica. 2009;11(1):393-403.

76. Williams C, Salter L, Jones G. The winter diet of the lesser horseshoe bat (*Rhinolophus hipposideros*) in Britain and Ireland. Hystrix, the Italian Journal of Mammalogy. 2010;22(1).

77. Zeale MRK, Butlin RK, Barker GLA, Lees DC, Jones G. Taxon‐specific PCR for DNA barcoding arthropod prey in bat faeces. Molecular Ecology Resources. 2011;11(2):236-44.

78. Zukal J, Gajdošík M. Diet of *Eptesicus serotinus* in an agricultural landscape. Vespertilio. 2012;16:357-63.
